# Novel Combined Training Approach Improves Sleep Quality but Does Not Change Body Composition in Healthy Elderly Women: A Preliminary Study

**DOI:** 10.1155/2017/8984725

**Published:** 2017-09-14

**Authors:** Thiago Matheus da Silva Sousa, Bruno Rodrigues, Marco Carlos Uchida, Olivia de Moraes Ruberti, Paulo Adriano Schwingel, Tânia Maria Gaspar Novais, Paula de Lourdes Lauande Oliveira, Fabiano de Jesus Furtado Almeida, Janaína Oliveira Bentivi Pulcherio, Bruno Bavaresco Gambassi

**Affiliations:** ^1^Physical Education Department, Ceuma University, São Luis, MA, Brazil; ^2^Faculty of Physical Education, University of Campinas (UNICAMP), Campinas, SP, Brazil; ^3^Human Performance Research Laboratory, University of Pernambuco, Petrolina, PE, Brazil; ^4^Health Science Graduate Programs, Federal University of Maranhão, São Luis, MA, Brazil

## Abstract

The aim of this study was to investigate the effects of a novel combined training protocol on sleep quality and body composition of healthy elderly women. The study sample consisted of 8 sedentary elderly individuals with mean (±SD) of 67 (±8) years of age, 96.0 (±7.8) mg/dL fasting blood glucose, 94.4 (±36.1) mg/dL triglycerides, 179.1 (±22.4) mg/dL total cholesterol, 57.2 (±15.7) mg/dL high-density lipoprotein (HDL), 103.1 (±25.2) mg/dL low-density lipoprotein (LDL), 125.3 (±8.4) mmHg systolic blood pressure, and 72.6 (±10.1) mmHg diastolic blood pressure. The training protocol consisted of resistance training exercises (approximately 18-minute duration) combined with aerobic exercises (approximately 26-minute duration), performed interspersed in the same session, for 8 weeks (3 times a week), with a 24-hour interval rest between each session. Continuous variables were expressed as the mean (±standard deviation) and the paired sample *t*-test compares baseline with final measurement. The results showed a significant improvement (*p* = 0.01) in quality of sleep (4.9 ± 1.5 versus 3.8 ± 1.8 for total PSQI index) without body significant improvements in the fat-free mass (59.9 ± 4.0 versus 60.5 ± 4.4; *p* = 0.20) and fat mass (40.1 ± 4.0 versus 39.5 ± 4.4; *p* = 0.20) in healthy elderly women. In this sense, the novel combined training proposed may be an effective alternative or adjunct to present therapies aimed at improving the sleep quality in this population.

## 1. Introduction

Aging is a multifactorial process which depends on changes occurring at the cellular and molecular level and is associated with a morphofunctional decline affecting all physiological systems with a high degree of interindividual variability [1, 2].

Problems with sleep are common during aging; usually, increasing light sleep and shortening REM sleep can occur [3, 4]. These alterations may cause damage to coronary artery, increase in sympathetic tone, and negative changes in blood pressure [5, 6]. In addition, sleep problems are related to depression, obesity, arthritis, stroke, and lung diseases [6]. Additionally, as we age, there is also an increase in body fat, along with structural and functional changes in the muscular system [7–9]. Poor sleep quality and increased body fat increase the risk of developing cardiovascular and metabolic diseases [5, 6, 10].

On the other hand, the practice of physical exercises may improve sleep quality and body composition of the elderly [11–20]. The findings of Gambassi et al. [12], Ferris et al. [13], and Cai et al. [14] have shown that both resistance training and aerobic training were effective in improving sleep quality.

Additionally, some studies have found positive effects of resistance training on bone mineral density, muscle mass, and body fat of elderly women [15, 16, 21]. Moreover, other investigations have demonstrated positive effects of aerobic training on autonomic parameters and hemodynamic profile of older women [22–24]. In view of these results, we may speculate that combining the two modalities might expand the benefits of physical exercise for elderly women.

In this sense, the studies undertaken by Bonardi et al. [11], Rossi et al. [18], Burich et al. [19], and Guedes et al. [20] have all demonstrated that combined training improves sleep quality and promotes significant positive changes in body composition of older women.

In addition to the benefits of combined training practice in older women [11, 18–20], the American College of Sports Medicine (ACSM) [25] recommends the weekly practice of aerobic exercises (3 to 5 days per week) associated with resistance exercises (2 to 3 days per week). However, by having to fulfill several personal tasks, many people cannot practice physical exercises every day. Given this, we designed a dynamic combined training approach, which intersperses resistance and aerobic training, thus addressing the time limitation of the subjects and increasing adherence of the participants. This approach may also reduce monotony and mirror more faithfully the typical daily physical needs of the elderly, which are made up of a mixture of aerobic with resistance activities.

To the best of our knowledge, this is the first study investigating the effects of 8 weeks of combined exercise training (resistance training and aerobic exercises interspersed in the same session) on sleep quality and body composition in healthy elderly women.

We hypothesize that the 8-week practice of this novel alternative combined training protocol may improve the variables investigated.

## 2. Materials and Methods

### 2.1. Experimental Approach to the Problem

In the first visit to the laboratory, a meeting was held to explain the research proposal for the elderly. Furthermore, an informal but detailed lecture described and carefully laid out the exercises that would be performed by the elderly. For a week, the physical training protocol was performed only with the purpose of familiarizing the elderly with the combined training protocol proposed. After these adjustments, measurements of sleep quality and body composition were carried out. The same set of measurements were undertaken after 8 weeks of combined exercise training practice.

All participants, after being properly informed about the study proposal, the procedures they would be undergo, and their potential risks and benefits, signed a free informed consent form. This study was approved by the Research Ethics Committee of the University Ceuma (number 813,886), as recommended by the Resolution 466/12 of the National Health Council.

### 2.2. Subjects

The sample was selected using convenience sampling. Exclusion criteria were as follows: being physically active and having any impairment in the musculoskeletal system and/or chronic degenerative diseases. To ensure participants met the criteria, all subjects were asked to undergo the following tests: fasting blood glucose, triglycerides, total cholesterol, low-density lipoprotein (LDL), and high-density lipoprotein (HDL).

The study sample consisted of 8 sedentary elderly individuals with mean (±SD) of 67.0 (±8.0) years of age, 96.0 (±7.8) mg/dL fasting blood glucose, 94.4 (±36.1) mg/dL triglycerides, 179.1 (±22.4) mg/dL total cholesterol, 57.2 (±15.7) mg/dL HDL cholesterol, 103.1 (±25.2) mg/dL LDL cholesterol, 125.3 (±8.4) mmHg systolic blood pressure, and 72.6 (±10.1) mmHg diastolic blood pressure.

### 2.3. Procedures

Data collection was performed by physical education undergraduate students from the Ceuma University. They had been previously trained by researchers and professors from the Physical Education Department at Ceuma University, Sao Luis, Brazil.

### 2.4. Sleep Quality Assessment

The Pittsburgh Sleep Quality Index (PSQI) was used to assess sleep quality. This questionnaire encompasses components of subjective quality, habitual efficiency, latency, disorders, medication use, duration, and diurnal dysfunctions. Each component was evaluated on a scale of zero to three points and given the same value, with three points marking the negative end of the scale. The sum of the values constitutes the total PSQI index; scores between 0 and 4 would indicate good sleep quality, 5 and 10 poor sleep quality, and above 10 sleep disturbance [26].

### 2.5. Evaluating Body Composition

Total body mass in kilograms (kg) and height in centimeters (cm) were measured using an anthropometric scale PL-200 (Filizola S.A. Pesagem e Automacão, São Paulo, SP, Brazil), with an accuracy of 50 g and 0.1 cm, and properly calibrated (NBR ISO/IEC 17025:2005). Following these evaluations, body composition (fat mass and fat-free mass) was measured through a bioimpedance analyser (Maltron BF-906, Maltron International Ltd., Essex, UK). All participants were hydrated and refrained from using any diuretics for 7 days and eating solid food for 4 hours prior to the test. They were advised to urinate prior to evaluation, to wear light clothing, and to not carry any metal objects during body composition evaluation [27].

### 2.6. Combined Training Program

This novel proposal of combined exercise training consisted of resistance training (approximately 18 min) combined with aerobic exercise (approximately 26 min), performed interspersed in the same session, for 8 weeks (three times a week) with a 24-hour interval rest between each session. We used values between 11 and 13 of Borg scale [28] for measuring the intensity of physical training for walking.

The resistance training protocol [15] consisted of running leg press at 180°, seated row, leg curl, bench press, abductor machine, push-down, adductor machine, and biceps curl, alternated with a segment of 3 sets of 8 repetitions maximum performed with a rest interval of 1-min between sets. In this sense, the load enabled the attainment of a specified number of repetitions per set until reaching concentric fatigue. The exercises were performed by isotonic contraction lasting 3 seconds for the concentric phase and 3 seconds for the eccentric phase (6 seconds per repetition) [25].

We used the combined training protocol proposed by Gambassi et al. [29]. This program consists of stimulating the neuromuscular and cardiovascular systems in an intercalated fashion from beginning to end of a session. As the proposal of the present study is very dynamic, our group named the program* dynamic combined training method.*

Each session included the following (in this sequence): 10 minutes of walking; leg press at 180°; 2 minutes of walking; seated row; 2 minutes of walking; leg curl; 2 minutes of walking; bench press; 2 minutes of walking; abductor machine; 2 minutes of walking; push-down; 2 minutes of walking; adductor machine; 2 minutes of walking; biceps curl; 2 minutes of walking.

The elderly women were divided into 3 groups before performing this sequence. All of them remained under the close supervision of students and the researcher. This division allowed for better load control, rest interval, and the application of the Borg scale [28].

### 2.7. Statistical Analyses

The data were processed and analyzed using PRISM software (GraphPad Software, San Diego, CA, USA, Release 5.01). Initially, descriptive statistics were applied to the Kolmogorov–Smirnov test and to Bartlett's criteria. Continuous variables were expressed as the mean (±standard deviation) and the *t*-test paired sample compared baseline (pre-) with final (post)measurement. All analyses are two-tailed, confidence intervals (CI) are exact, and *p* values were calculated using a significance level of 5%. The magnitude of the differences in variables was calculated from the effect size [30].

## 3. Results and Discussion

**Figure 1:**
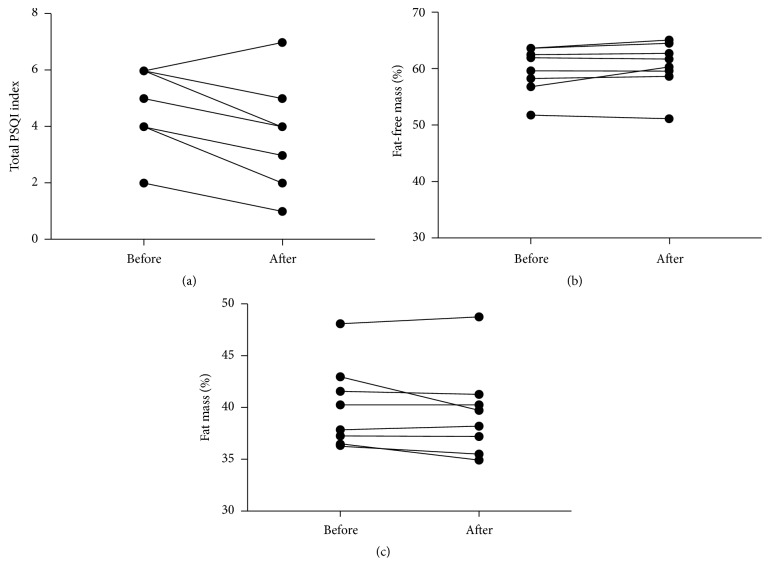
Changes in quality of sleep (a), fat-free mass (%) (b), and fat mass (%) (c) for each elderly woman before (in baseline) and after 8 weeks of a dynamic combined training program.

Significant differences in the quality of sleep (*p* = 0.01; Δ = −1.1) were determined by comparing baseline and post-combined training results. In relation to this variable, large negative effect sizes were verified after 8 weeks of combined training [*p* = 0.01; *d*  = −1,2 (95% CI −1,95 to −0,29)]. Changes were observed in fat-free mass (%) (*p* = 0.20; Δ = 0.6) and in body fat (%) (*p* = 0.20; Δ = −0.6). However, we did not find any significant differences between these variables (see Table 1 and Figure 1).

The findings of the present study partially confirm our hypothesis, since the dynamic combined training program (resistance training exercises combined with aerobic exercises interspersed in the same session) provided significant improvement in the sleep quality among healthy elderly women.

These findings emphasize the importance of physical training as a nonpharmacological strategy for improving sleep quality. In line with our results, Bonardi et al. [11] have demonstrated benefits of combined training (strength training plus aerobic exercise) on the quality of sleep in hypertensive elderly women. The combined training protocol adopted by these researchers was carried out for 10 weeks on alternate days for a total of 30 uninterrupted sessions. The protocol consisted of performing strength training with 50 to 60% of one repetition maximum (RM) intensity (one circuit with leg press at 45°, bench press, extensor bench, handle front, flexor bench-sitting, upright row, plantar flexion, seated row, and abdominals from 1st to 4th week, with a progression to 2 circuits between 5th and 10th week) plus aerobic exercises performed on treadmill for 20 minutes, from the 1st to the 4th week, with a progression to 30 minutes from the 5th to the 10th week.

Studies investigating the effects of combined training on sleep quality of the elderly remain scarce in the literature; however, some research has found that aerobic or resistance training improves sleep quality in the elderly and in postmenopausal women. Gambassi et al. [12] have compared elderly people practicing moderate-intensity resistance training for 12 weeks to sedentary elderly and found that the active population presented better sleep quality. Additionally, Ferris et al. [13] have shown that resistance training (circuit training method) performed for 3 months resulted in a significant improvement in sleep quality in the elderly.

As to aerobic training of moderate to high intensity, with a frequency of 3 times per week for 10 weeks, Cai et al. [14] have found and increase in melatonin and a significant improvement in sleep quality of postmenopausal women after 10 weeks of training. Each session of the exercise program of the study was comprised of warm-up with 10 to 15 minutes of stretching exercises for major muscles, step aerobics exercise in which the target HR was reached in the first 5 to 10 minutes and held for another 30 to 35 minutes, balance and cool-down for 10 to 15 minutes, and stretching and relaxation for 10 to 15 minutes. Additionally, Bonardi et al. [11] have also demonstrated positive effects on the sleep quality of elderly hypertensive patients after 10 weeks of aerobic training (exercises performed on the treadmill for 20 minutes from the 1st to the 4th week, with progression to 30 minutes from the 5th to the 10th week).

The findings of Bonardi et al. [11], Gambassi et al. [12], Ferris et al. [13], and Cai et al. [14] have shown that combined, resistance, or aerobic training was effective in improving sleep quality. Theories of energy conservation, thermoregulation, and body restoration are among the hypotheses to explain the mechanisms of sleep quality improvement through the practice of physical exercises [31]. However, more randomized controlled trials are needed to account for the mechanisms underlying exercise-induced improvements in sleep quality in the elderly.

As previously mentioned, in addition to the loss of strength during the aging process, negative changes affect body composition [7–9]. In the present study, we observed a reduction of fat percentage and increase in fat-free mass; however, no significant differences were found for these variables. In line with the results of the present study, Campos et al. [32] have not found any significant changes in body composition after the 12-week practice of 2 models of training (treadmill aerobic training plus strength training and strength training followed by aerobic treadmill training) performed by active elderly women. In both combined training protocols of their study [32], the traditional periodization was used (decreasing the volume and increasing the intensity over the weeks).

Unlike our study, the research carried out by Rossi et al. [18] has demonstrated significant benefits on body composition with increased fat-free mass and decreased fat mass in elderly women. These changes were observed after 16 weeks of combined training (27 min resistance training session plus 30 min aerobic training session performed in the same day). The intensity of the resistance training was adjusted every 4 weeks with increases in the RM zone. Concerning aerobic training, the intensity was adjusted after 4 weeks through the critical velocity.

Similarly, after performing the combined training, Burich et al. [19] have observed a significant reduction in body fat and in the waist-to-hip ratio in elderly. The combined training consisted of 20 minutes of cycling at 65% of heart rate reserve, followed by 20 minutes of strength training (3 sets of 15 RM in weeks 1 to 4; 3 sets of 12 RM in weeks 4 to 8; and 3 sets of 10 RM in weeks 8 to 12) three times a week for 12 weeks. Regarding the increase of muscular mass, the study corroborates the research undertaken by Rossi et al. [18]; Guedes et al. [20] have found an increase in the size of the vastus lateralis muscle after 8 weeks of combined training (2 sessions per week, consisting of 1 session of aerobic training and 1 session of resistance training lasting no more than 30 minutes a day, performed on different days).

The studies undertaken by Rossi et al. [18], Burich et al. [19], and Guedes et al. [20] have demonstrated that combined training promotes significant positive changes in the body composition of the elderly persons. However, we could not observe these changes in the present study, and our small sample might account for this discrepancy. A study conducted by Campos et al. [32] with a small number of participants (combined training with 5 participants) did not find any significant changes in body fat. Lack of dietary control may be another possible explanation for the results associated with body composition in our study.

The findings of the present study may inform strength and conditioning professionals on the possibility of achieving benefits on the quality of sleep of elderly women through a novel combined training approach.

The protocol used in this study (resistance and aerobic exercises interspersed in the same session) was not used in any of the other studies mentioned above. However, other training protocols have demonstrated positive effects on the variables investigated in our research [11, 18–20]. We should stress that the 24-session duration of our protocol presented benefits similar to studies using longer intervention time [11, 18–20]. Thus, the quality of sleep may be improved with our 8-week combined training protocol.

Our study has some limitations worth commuting upon. In this study, the absence of a control group, the lack of randomization, the small sample size, and the lack of diet control may be regarded as limitations. However, as a preliminary study, we raise issues for novel areas of research. In addition, we did not have the resources to assess aerobic capacity (VO2max) and perform exams at baseline after the combined training program. We found it difficult to have the elderly performing the 1 repetition maximum test in our previous research, since they manifested fear and the evaluation was underestimated. Thus, our groups have always adopted maximum repetitions protocols.

## 4. Conclusions

We conclude that the dynamic combined training program (resistance training and aerobic exercises interspersed in the same session) may provide benefits on the quality of sleep of elderly. However, our findings need further investigation, and adding randomized controlled trial would be required to lend support to our dynamic approach as an effective alternative or adjunct to present therapies aimed at improving sleep in this population

## Figures and Tables

**Table 1:** Sleep quality and body composition before (in baseline) and after 8 weeks of a dynamic combined training program (*n* = 8).

Variables	Test moments		*p* values
	Before (*n* = 8) Mean ± SD	After (*n* = 8) Mean ± SD	
*Sleep quality*			
Total PSQI index	4.9 ± 1.5	3.8 ± 1.8^*∗*^	0.01
*Body composition*			
Fat-free mass (%)	59.9 ± 4.0	60.5 ± 4.4	0.20
Fat mass (%)	40.1 ± 4.0	39.5 ± 4.4	0.20

SD: standard deviation of the mean; kg: kilograms; ^*∗*^significant difference between the pre- and posttest results.

## References

[1] de Moraes E. N., de Moraes F. L., Lima S. D. P. P. (2010). Características biológicas e psicológicas do envelhecimento. *Revista Medicina Minas Gerais*.

[2] Coelho Júnior H. J., Aguiar S. D. S., Gonçalves I. D. O. (2015). Sarcopenia is associated with high pulse pressure in older women. *Journal of Aging Research*.

[3] Cooke J. R., Ancoli-Israel S. (2011). Normal and abnormal sleep in the elderly. *Handbook of Clinical Neurology*.

[4] King C. R., Knutson K. L., Rathouz P. J., Sidney S., Liu K., Lauderdale D. S. (2008). Short sleep duration and incident coronary artery calcification. *Journal of the American Medical Association*.

[5] Yuksel M., Yildiz A., Demir M. (2014). Effect of sleep quality on hemodynamic response to exercise and heart rate recovery in apparently healthy individuals. *Clinical and Investigative Medicine*.

[6] Foley D., Ancoli-Israel S., Britz P., Walsh J. (2004). Sleep disturbances and chronic disease in older adults: results of the 2003 national sleep foundation sleep in america survey. *Journal of Psychosomatic Research*.

[7] Faulkner J. A., Larkin L. M., Claflin D. R., Brooks S. V. (2007). Age-related changes in the structure and function of skeletal muscles. *Clinical and Experimental Pharmacology and Physiology*.

[8] Lang T., Streeper T., Cawthon P., Baldwin K., Taaffe D. R., Harris T. B. (2010). Sarcopenia: etiology, clinical consequences, intervention, and assessment. *Osteoporosis International*.

[9] Narici M. V., Maffulli N. (2010). Sarcopenia: characteristics, mechanisms and functional significance. *British Medical Bulletin*.

[10] Jung U. J., Choi M. S. (2014). Obesity and its metabolic complications: the role of adipokines and the relationship between obesity, inflammation, insulin resistance, dyslipidemia and nonalcoholic fatty liver disease. *International Journal of Molecular Sciences*.

[11] Bonardi J. M. T., Lima L. G., Campos G. O. (2016). Effect of different types of exercise on sleep quality of elderly subjects. *Sleep Medicine*.

[12] Gambassi B. B., Almeida F. J. F., Sauaia B. A. (2015). Resistance training contributes to variability in heart rate and quality of the sleep in elderly women without comorbidities. *Journal of Exercise Physiology Online*.

[13] Ferris L. T., Williams J. S., Shen C. L., OKeefe K. A., Hale K. B. (2005). Resistance training improves sleep quality in older adultsa pilot study. *Journal of Sports Science and Medicine*.

[14] Cai Z.-Y., Chen K. W.-C., Wen H.-J. (2014). Effects of a group-based step aerobics training on sleep quality and melatonin levels in sleep-impaired postmenopausal women. *Journal of Strength and Conditioning Research*.

[15] Gambassi B. B., Rodrigues B., Feriani D. J. (2016). Effects of resistance training of moderate intensity on heart rate variability, body composition, and muscle strength in healthy elderly women. *Sport Sciences for Health*.

[16] Huang S. W., Ku J. W., Lin L. F., Liao C. D., Chou L. C., Liou T. H. (2017). Body composition influenced by progressive elastic band resistance exercise of sarcopenic obesity elderly women: a pilot randomized controlled trial. *European Journal of Physical and Rehabilitation Medicine*.

[17] Park S.-M., Kwak Y.-S., Ji J.-G. (2015). The effects of combined exercise on health-related fitness, endotoxin, and immune function of postmenopausal women with abdominal obesity. *Journal of Immunology Research*.

[18] Rossi F. E., Fortaleza A. C. S., Neves L. M. (2016). Combined training (aerobic plus strength) potentiates a reduction in body fat but demonstrates no difference on the lipid profile in postmenopausal women when compared with aerobic training with a similar training load. *Journal of Strength and Conditioning Research*.

[19] Burich R., Teljigović S., Boyle E., Sjøgaard G. (2015). Aerobic training alone or combined with strength training affects fitness in elderly: randomized trial. *European Journal of Sport Science*.

[20] Guedes J. M., Bortoluzzi M. G., Matte L. P. (2016). Effects of combined training on the strength, endurance and aerobic power in the elderly women. *Revista Brasileira de Medicina do Esporte*.

[21] Kemmler W., Engelke K., Lauber D., Weineck J., Hensen J., Kalender W. A. (2002). Exercise effects on fitness and bone mineral density in early postmenopausal women: 1-Year EFOPS results. *Medicine and Science in Sports and Exercise*.

[22] Stein P. K., Ehsani A. A., Domitrovich P. P., Kleiger R. E., Rottman J. N. (1999). Effect of exercise training on heart rate variability in healthy older adults. *American Heart Journal*.

[23] Carvalho P., Barros G., Melo T., Santos P., Oliveira G., DAmorim I. (2013). Efeito dos treinamentos aeróbio, resistido e concorrente na pressão arterial e morfologia de idosos normotensos e hipertensos. *Revista Brasileira de Atividade Física and Saúde*.

[24] Perini R., Fisher N., Veicsteinas A., Pendergast D. R. (2002). Aerobic training and cardiovascular responses at rest and during exercise in older men and women. *Medicine and Science in Sports and Exercise*.

[25] American College of Sports Medicine (2007). *Diretrizes do ACSM para os Testes de Esforço e sua Prescrição*.

[26] Buysse D. J., Reynolds C. F., Monk T. H., Berman S. R., Kupfer D. J. (1989). The Pittsburgh Sleep Quality Index: a new instrument for psychiatric practice and research. *Psychiatry Research*.

[27] Lukaski H. C. (1999). Requirements for clinical use of bioelectrical impedance analysis (BIA). *Annals of the New York Academy of Sciences*.

[28] Borg G. (1998). *Borgs Perceived Exertion and Pain Scales*.

[29] Gambassi B. B., Uchida M. C., Sousa T. M. S., Schwingel P. A., Pulcherio J. O. B., Almeida F. J. F. (2017). Effects of a new combined training approach on components of the functional autonomy of healthy elderly women. *Journal of Exercise Physiology Online*.

[30] Cohen J. (1988). *Statistical Power Analysis for the Behavioral Sciences*.

[31] Martins P. J. F., Mello M. T. D., Tufik S. (2001). Exercício e sono. *Revista Brasileira de Medicina do Esporte*.

[32] Campos A. L. P., Del Ponte L. D. S., Cavalli A. S., Afonso M. D. R., Schild J. F. G., Reichert F. F. (2013). Efeitos do treinamento concorrente sobre aspectos da saúde de idosas. *Revista Brasileira de Cineantropometria e Desempenho Humano*.

